# Bidirectional relationship between epigenetic age and brain health events

**DOI:** 10.21203/rs.3.rs-4378855/v1

**Published:** 2024-06-25

**Authors:** Cyprien Rivier, Natalia Szejko, Daniela Renedo, Santiago Clocchiatti-tuozzo, Shufan Huo, Adam de Havenon, Hongyu Zhao, Thomas Gill, Kevin Sheth, Guido Falcone

**Affiliations:** Yale School of Medicine; Medical University of Warsaw; Yale School of Medicine; Yale School of Medicine; Yale School of Medicine; Yale University School of Medicine; Yale University; Yale School of Medicine; Yale University School of Medicine; Yale University

## Abstract

Chronological age offers an imperfect estimate of the molecular changes that occur with aging. Epigenetic age, which is derived from DNA methylation data, provides a more nuanced representation of aging-related biological processes. This study examines the bidirectional relationship between epigenetic age and the occurrence of brain health events (stroke, dementia, and late-life depression). Using data from the Health and Retirement Study, we analyzed blood samples from over 4,000 participants to determine how epigenetic age relates to past and future brain health events. Study participants with a prior brain health event prior to blood collection were 4% epigenetically older (beta 0.04, SE 0.01), suggesting that these conditions are associated with faster aging than that captured by chronological age. Furthermore, a one standard deviation increase in epigenetic age was associated with 70% higher odds of experiencing a brain health event in the next four years after blood collection (OR 1.70, 95%CI 1.16–2.50), indicating that epigenetic age is not just a consequence but also a predictor of poor brain health. Both results were replicated through Mendelian Randomization analyses, supporting their causal nature. Our findings support the utilization of epigenetic age as a useful biomarker to evaluate the role of interventions aimed at preventing and promoting recovery after a brain health event.

Age remains the principal risk factor for neurodegenerative conditions^1^ and the most substantial non-modifiable determinant for cerebrovascular disease, posing significant challenges to understanding the complex interplay of biological and molecular aging processes with disease risk^2^. Despite chronological age serving as a conventional marker, recent advancements have introduced more sophisticated measures of aging. Central to these innovations are epigenetic clocks, a novel approach based on the analysis of DNA methylation patterns at CpG sites^3^. This methylation process chemically alters DNA molecules, thereby modulating gene expression without changing the DNA sequence. In contrast to the DNA sequence, which remains largely unchanged throughout life, DNA methylation exhibits a degree of plasticity, allowing for changes in response to diverse lifestyle and environmental exposures, including established cardiovascular risk factors^4^.

Epigenetic clocks, derived from weighted aggregation of methylation across select CpG sites, echo the principles of polygenic risk scores, offering a quantifiable measure of biological age^5^. The selection of CpG sites and their integration into a singular biological age metric is informed by robust statistical models trained on specific outcomes, ranging from chronological age to more complex phenotypes associated with healthspan and lifespan. This approach has led to the development of various epigenetic clocks. Initially, these clocks were calibrated on chronological age^6–10^, but subsequent iterations have focused on broader phenotypes, such as time-to-death^11^ or clinical parameters linked to morbidity and mortality^3^. Notably, some epigenetic clocks, such as the PhenoAge^3^, GrimAge^11^, and Zhang^12^ clocks have demonstrated a superior ability to predict mortality and various health outcomes, significantly surpassing the predictive power of chronological age.

The pursuit of health and longevity is fundamentally tied to the preservation of a healthy brain. In the context of an aging global population, the imperative to sustain brain health becomes paramount, especially given the increased prevalence and incidence of neurological disorders, now the leading cause of disability-adjusted life years worldwide^13^. Among aging-related brain diseases, stroke, dementia, and late-life depression have the highest prevalence and incidence^14^, significantly impacting global brain health due to their disruptive effects on normal brain function. These conditions are closely related, sharing risk factors such as smoking, diet, physical activity, and socio-economic health determinants^15–19^, which are also known to influence epigenetic clocks.^4^ Furthermore, stroke, dementia, and late-life depression can act as risk factors for each other, creating a complex web of interacting health problems^20,21^. Finally, the occurrence of late-life depression has been shown to be associated with cerebral small vessel disease, aligning it with stroke and dementia from a pathophysiological perspective^22,23^. This intricate relationship has given rise to the view that these conditions should not be treated as isolated outcomes, but as interconnected components of a broader aging process that requires a comprehensive approach^24,25^. To promote healthy aging, it is thus necessary to deepen our understanding of the relationship between brain health and the systemic manifestations of the aging process.

Given the growing interest in understanding the aging process beyond chronological age and growing importance of brain health as a determinant of healthy aging, we tested the hypothesis that brain health events accelerate epigenetic aging, and conversely, that accelerated epigenetic aging increases the risk of brain health events. Given that the study of DNA methylation in brain health is still in its early stages, research in this field is limited and often involves small sample sizes. To address this, we conducted our analyses using the Health and Retirement Study, a large longitudinal study of older adults that is representative of the U.S. population. The collection of DNA methylation data in 2016 provided a unique opportunity to assess the impact of past brain health events as well as the future risk of such events in relation to epigenetic age. To evaluate the hypothesized bidirectional relationships, we used both traditional epidemiological associations and a genetic mendelian randomization (MR) framework. By leveraging genetic variants as instrumental variables, MR enabled us to support the causality of these associations with a higher level of evidence compared to observational analyses alone^26,27^.

## RESULTS

### Cohort characteristics

**Figure 1:**
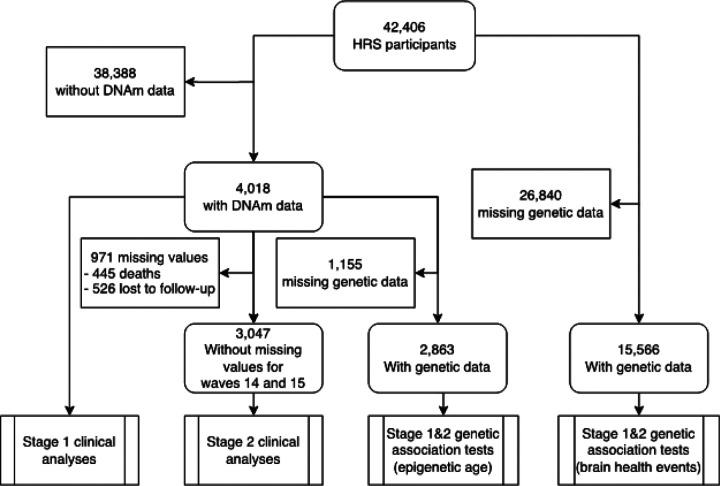
Flowchart

The HRS enrolled 42,233 participants between 1992 and 2016. Of these, 4,018 provided blood samples in 2016 and were included in our analyses (Figure 1). Comparison of baseline characteristics between the complete HRS cohort and the subset with DNA methylation (DNAm) data can be found in Supplementary Table 1. The baseline characteristics of the studied population are presented in Table 1 (mean age: 70, 58% females). The average age at DNAm data acquisition was 70 years, 58% were females, 17% were Blacks, and 5% were Hispanics.

### First stage: history of brain health events and epigenetic age

#### Observational analyses

Of the 4,018 participants included in this cross-sectional analysis at the time of blood sample collection in 2016, 342 (8.5%) had a stroke, 298 (7.4%) had dementia, and 322 (8.0%) already had a late-life major depressive episode prior to DNAm acquisition. This resulted in 806 (20.1%) participants with a history of at least one brain health event, including 127 (3.2%) with two events and 13 (0.3%) with all three events. In multivariable linear regression adjusting for age, sex, race/ethnicity, cardiovascular risk factors (BMI, smoking status) and comorbidities (hypertension, diabetes, heart attack, coronary artery disease, angina, congestive heart failure), brain health events were associated with a 4% increase (beta = 0.04, SD = 0.01, p=0.002) in mean normalized epigenetic age (Figure 4 and Table 2). This association was strengthened when only adjusting for age, sex and race/ethnicity, with an 8% increase (beta = 0.08, SD = 0.01, p<0.001) in mean epigenetic age.

In secondary analyses that considered each brain health event type separately, a history of stroke was associated with a 6% increase in epigenetic age (beta = 0.06, SD = 0.02, p=0.001 - Figure S2 and Table S7) after adjusting for demographics, risk factors, and comorbidities. Similarly, a history of dementia was associated with a 4% increase (beta = 0.04, SD = 0.02, p=0.035 - Figure S3 and Table S9). A history of late-life major depressive disorder was not associated with an increase in epigenetic age in the fully adjusted model (beta= 0.01, SD = 0.02, p=0.673 - Figure S4 and Table S11). Also, a history of either stroke or dementia was associated with a 4% increase in mean epigenetic age (beta= 0.04, SD = 0.01, p=0.003 - Figure S1 and Table S5).

#### Sensitivity analysis: late-life depression ascertained with a different age threshold

Given the existing variation in the age cutoff used to define late-life depression, in sensitivity analyses we considered an age threshold of 60 instead of 65 at the first major depressive episode. Out of 4,018 participants, 583 (14.5%) had a late-life depression prior to DNAm acquisition and 1,014 (25.2%) had a history of at least one brain health event. In multivariable linear regression adjusting for age, sex and race/ethnicity, brain health events were associated with an 8% increase (beta = 0.08, SD = 0.01, p<0.001) in mean normalized epigenetic age. After adjusting for cardiovascular risk factors and comorbidities as well, a history of brain health events was associated with a 5% increase (beta = 0.05, SD = 0.01, p<0.001) in mean epigenetic age (Table S13).

#### Mendelian randomization analyses

**Figure 2:**
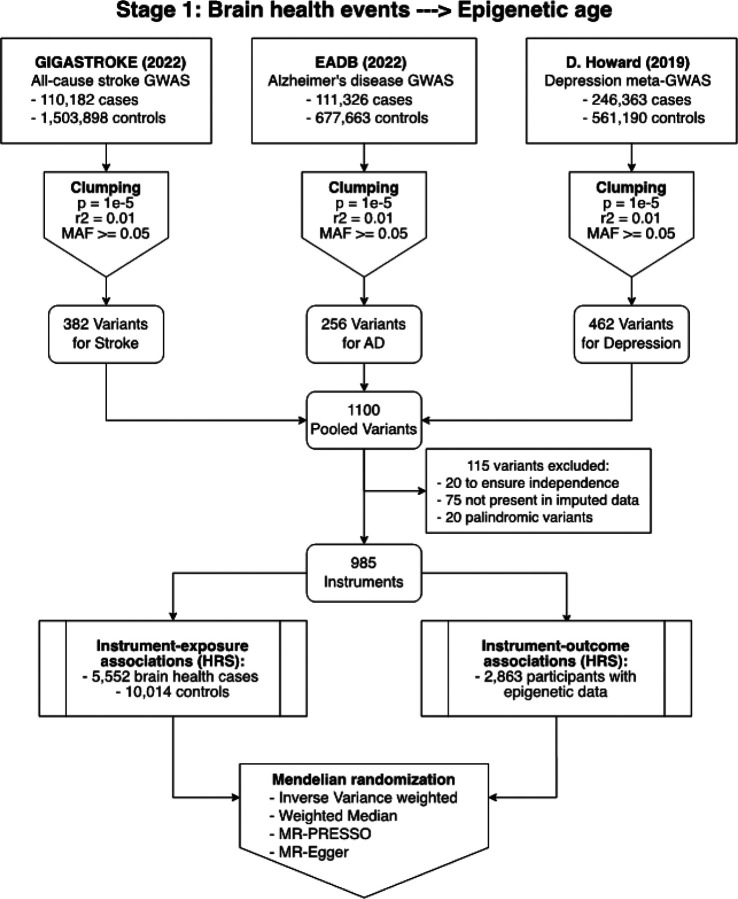
Flowchart of Stage 1 genetic analyses

Several different MR analyses (Figure 2) confirmed a positive association between genetically determined brain health events and accelerated epigenetic aging. In the primary analysis using 985 independent genetic instruments for brain health events and the inverse variance weighted MR method, genetically determined brain health events were associated with a 11% increase in mean epigenetic age (beta = 0.11, SD = 0.03, P < 0.001 – Table 3). The weighted median and MR-Egger methods, more conservative analytical approaches that are more robust to horizontal pleiotropy, yielded similar results, with genetically determined brain health events being associated, respectively, with 8% (beta = 0.8, SD = 0.04, P = 0.052) and 10% (beta = 0.1, SD = 0.04, P = 0.01) increases in epigenetic age. The MR-PRESSO global test and the MR-Egger Intercept did not suggest the presence of pleiotropy.

### Second stage: epigenetic age and subsequent risk of brain health events

#### Observational analyses

Of the 4,018 participants with DNAm data, 806 (20.1%) had a history of brain health events before 2016 and 245 (6.1%) were missing data after the DNAm acquisition in 2016 (waves 14 and 15), including 116 (2.9%) who died and 129 (3.2%) who were lost to follow-up (Figure 1). Of the 2,967 participants included in the prospective analysis, 81 (2.7%) developed a stroke, 100 (3.4%) developed dementia and 95 (3.2%) developed a late-life major depressive disorder. This resulted in 261 (8.8%) participants developing at least one brain health event over the 4 years of follow-up, including 15 (0.5%) developing two. In multivariable logistic regression adjusting for demographics (age, sex and race/ethnicity), one SD increase in epigenetic age was associated with a 70% increase (OR = 1.70, 95%CI: 1.16–2.50) in the odds of brain health events (Figure 4 and Table 2). The inclusion of cardiovascular risk factors (BMI, smoking status) and comorbidities (hypertension, diabetes, heart attack, coronary artery disease, angina, and congestive heart failure) in this analysis is subject to debate. These factors are known to influence methylation changes and might be implicitly reflected in the baseline estimation of epigenetic age. Therefore, adjusting for these variables could potentially constitute an overadjustment. Nevertheless, a model that additionally accounted for these factors, alongside demographics, indicated that a one SD increase in epigenetic age was still associated with a 48% increase in the odds of brain health events (OR = 1.48, 95% CI: 0.99–2.21 – Table 2).

In secondary analyses, we observed that epigenetic age acceleration was associated with an increased likelihood of experiencing a combined outcome of stroke and dementia. This association was also observed when stroke and dementia were analyzed separately. However, no such association was found with late-life depression. Specifically, we found a 112% increase in the odds of developing either stroke or dementia (OR = 2.12, 95% CI: 1.35–3.32 – see Figure S1 and Table S6) for each one SD increase in epigenetic age, after adjusting for demographics. Similar results were obtained when considering stroke (OR = 2.12, 95% CI: 1.12–4.04 – see Figure S2 and Table S8) and dementia (OR = 1.98, 95% CI: 1.10–3.56 – see Figure S3 and Table S10) individually. However, for late-life depression, the association was entirely non-significant (OR = 0.80, 95% CI: 0.43–1.52 – see Figure S4 and Table S12).

#### Mendelian randomization analyses

**Figure 3:**
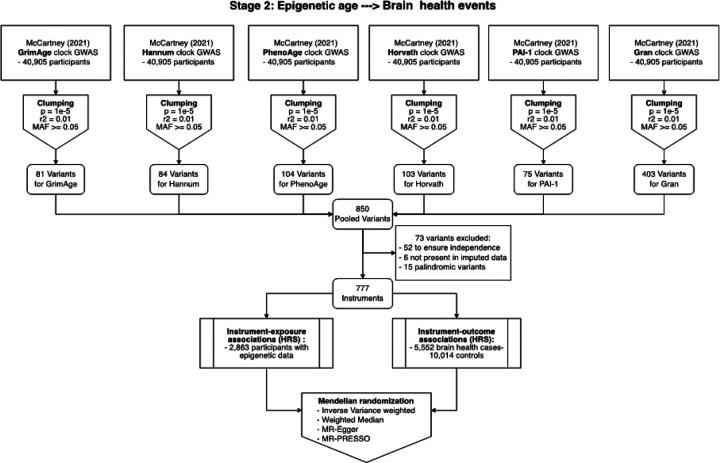
Flowchart of Stage 2 genetic analyses.

**Figure 4:**
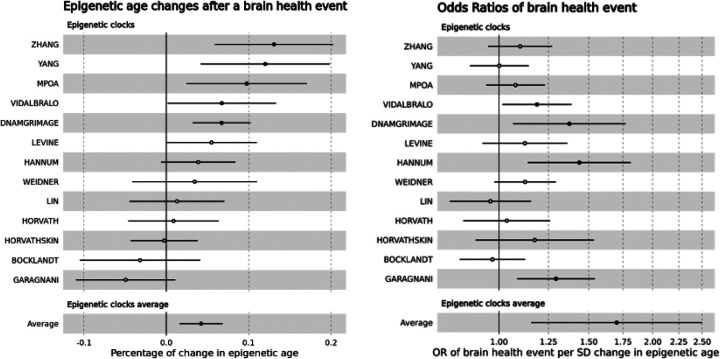
Associations between epigenetic age and brain health events (stroke, dementia, late-life depression). A. Cross-sectional analysis: percentage of change in epigenetic ages following a brain health event after adjusting for chronological age, sex, race and ethnicity, hypertension, diabetes, smoking, BMI, history of heart attack, coronary artery disease, angina, or congestive heart failure. B. Longitudinal analysis: Odds Ratios of brain health events per one standard deviation increase in epigenetic age adjusting for chronological age, sex, and race and ethnicity.

Several different MR approaches (Figure 3) confirmed a positive association between genetically determined epigenetic age and higher odds of brain health events. In the primary analysis using 777 independent genetic instruments and the inverse variance weighted MR method, one SD increase in genetically determined epigenetic age was associated with 15% higher odds of brain health events (OR = 1.15, 95%CI: 1.06–1.25 – Table 3). The weighted median method yielded similar results (OR = 1.15, 95%CI: 1.00–1.31), as well as the MR Egger method (OR = 1.15, 95%CI: 1.00–1.31). The MR-PRESSO global test as well as the Egger intercept were not significant, indicating no substantial pleiotropy.

#### Sensitivity analysis: late-life depression ascertained with a different age threshold

We replicated the observational analyses with late-life depression ascertained using an age threshold of 60 instead of 65 at the first major depressive episode. Out of the 2,779 participants included in the prospective analysis, 121 (4%) developed a late-life depressive disorder and 269 (10%) developed at least one brain health event over the 4 years of follow-up. In multivariable logistic models adjusting for demographics, one SD increase in epigenetic age was associated with a 57% increase (OR = 1.57, 95%CI: 1.07–2.31) in the odds of brain health events.

#### Sensitivity analysis: exclusion of people missing any follow-up waves

We replicated the observational analyses excluding those participants missing data for any of the waves 14 and 15, as opposed to only excluding participants missing data for both of the two waves. Of the 4,018 participants with DNAm data, 804 (20%) had a history of brain health event, 245 (6%) died and 394 (10%) were missing data for any of the waves 14 and 15, so this analysis included 2,573 participants. Of these, 79 (3%) developed a stroke, 75 developed dementia (3%), and 78 (3%) developed a late-life major depressive disorder. We observed a similar trend as in the primary analysis with a 1SD increase in epigenetic age leading to a 78% (OR = 1.78, 95%CI: 1.16 −2.72, Table S15) increase in the odds of brain health events after accounting for demographics.

## DISCUSSION

In this two-stage epigenetic study within the Health and Retirement Study, we identified a significant bidirectional relationships between epigenetic aging and brain health events. In the first stage, the cross-sectional analysis revealed an association between a history of brain health events and accelerated epigenetic age. Specifically, patients with a prior history of stroke, dementia, or late-life depression exhibited a statistically significant increase in mean normalized epigenetic age, findings that remained robust after adjusting for a range of covariates. This association was further confirmed through Mendelian Randomization analyses, suggesting a causal linkage. In the second stage, the prospective cohort analysis revealed that individuals with an accelerated epigenetic age were at a substantially higher risk of developing brain health events. This association persisted after comprehensive adjustments for confounders and was also observed in Mendelian Randomization analyses, again providing evidence for a causal relationship. These findings underscore the reciprocal influence between accelerated aging and the manifestation of brain health events, enhancing our comprehension of this complex interplay.

Mounting evidence points to the importance of epigenetic age as a more accurate indicator of true biological aging compared to chronological age^3,28^. Numerous studies have established that DNA methylation predicts all-cause mortality more accurately than chronological age alone^29–32^. This predictive ability has been first studied using epigenetic data from specific tissues, where methylation patterns are closely linked to disease development. For instance, accelerated epigenetic aging in the dorsolateral prefrontal cortex is associated with increased amyloid accumulation and cognitive decline in Alzheimer’s disease^33^. Similarly, the progression of osteoarthritis and obesity is reflected in the accelerated methylation patterns of cartilage^34^ and liver tissues^35^, respectively. Given the challenges and risks associated with tissue-specific sample collection, whole blood samples have become increasingly utilized for determining epigenetic age^28^. This approach has been validated, showing a high correlation between epigenetic age derived from whole blood and that from specific tissues, making it a reliable proxy for general epigenetic age assessment^3^. Subsequently, blood-derived epigenetic age acceleration has been linked to the occurrence of various conditions including cancer^36–39^, cardiovascular and coronary heart diseases^3^, Parkinson’s disease^40^ and frailty^41,42^. In addition, key risk factors such as high blood pressure^43^, BMI^35^, triglycerides^3^, or glucose levels^3,43^, as well as smoking^3^ and low physical activity^3,43^ have been shown to accelerate aging-related epigenetic modifications. These findings emphasize the influence of environmental factors and the dynamic nature of DNA methylation status. Finally, at a cellular level, DNA methylation clocks have been connected to three of the nine recognized hallmarks of aging^44^: nutrient sensing, mitochondrial function, and stem cell composition, highlighting their integral role in characterizing the aging process^45^.

This study adds important new evidence to epigenetic aging research by focusing on a broad observational outcome related to brain health. Stroke, dementia, and late-life depression, the most common aging-related brain conditions, are intricately linked. They share overlapping risk factors, including smoking, diet, physical activity, and socio-emotional health determinants, which contribute to the occurrence of all three^15–19^ and a common small vessel disease pathophysiology^22,23^. Furthermore, the occurrence of one condition markedly increases the likelihood of developing the others: a history of depression heightens the risk of stroke^46^ and dementia^47–49^; stroke raises the chances of subsequent dementia^21^ or depression^50^; and dementia itself is a risk factor for both hemorrhagic stroke^51^ and depression^52^. This intricate interplay has led to the perspective that these conditions should not be examined in isolation, but rather collectively, as distinct yet connected manifestations of a broader brain health aging process^24,25^. Our findings lend substantial support to this viewpoint. We demonstrate that an acceleration in the body’s epigenetic aging process significantly increases the risk of developing stroke or dementia, but not late-life depression. Because the pace of epigenetic aging can be slowed by lifestyle changes such as diet and exercise^43^, our results suggest that taking care of our body as we get older is a potentially effective way of preventing brain health events. Moreover, our study reveals that stroke and dementia not only result from, but also contribute to, a general acceleration of epigenetic aging, as evidenced by blood-derived methylation changes. These results underscore the systemic nature of these conditions, suggesting that they should be considered comprehensively, rather than as pure neurological or psychiatric disorders.

Our study also provides important evidence suggesting that the association between epigenetic aging and brain health are causal, as demonstrated by the results of our MR analyses. MR is an epidemiological method that leverages DNA sequence variants as instrumental variables, offering a powerful means to deduce potential causal links between exposures and outcomes^26,27^. By employing genetic variants that are randomly assigned during meiosis and remain constant throughout an individual’s life, MR effectively acts as a form of natural randomization. This approach is particularly valuable as it helps to counteract confounding by environmental factors and reverse causation, which are prevalent sources of bias in observational studies. Consequently, MR serves as a valuable tool, complementing observational studies by adding a layer of evidence to suggest the causal nature of observed relationships^53^. However, it is important to acknowledge that MR does not replace randomized controlled trials, which are still the gold standard for establishing causal associations. MR provides a crucial bridge in the hierarchy of scientific proof, particularly in scenarios where conducting trials is impractical or unethical.

Our findings pave the way for new research directions, particularly in exploring how epigenetic clocks can aid in the early detection of individuals at elevated risk of poor brain health. Currently, observational risk scores and polygenic risk scoring are widely recognized methods for categorizing individuals into different risk groups^54^. Our study suggests that epigenetic clocks could fulfill a similar role and could potentially be integrated with other risk scores to enhance the precision in predicting those most susceptible to brain health events. This combined approach could significantly facilitate early intervention strategies. Furthermore, there is potential for therapeutic interventions focused on modulating the epigenetic aging process itself, with the goal of preventing aging-related observational events. Recent research in mice has shown that DNA methylation clocks can be reversed through epigenetic reprogramming, leading to notable increases in life expectancy^55^. This underscores the profound influence of epigenetic modifications on the aging process as a whole. Such breakthroughs open possibilities for the development of targeted treatments that not only manage but also proactively mitigate the risks of aging-related neurological conditions by addressing their underlying epigenetic mechanisms.

The primary strength of our study is the utilization of the Health and Retirement Study, which is among the largest and best characterized cohorts with DNA methylation data. Acquiring DNA methylation data is often a costly endeavor, leading to smaller datasets that typically require integration with other datasets to reach sufficient power^11^. The Health and Retirement Study’s substantial size, combined with its demographic representativeness of the US population, significantly bolsters the generalizability of our findings to older Americans. Additionally, the application of MR analyses enabled us to strengthen our observational results, providing a more compelling argument for the causal nature of the relationships we identified. However, our study is not without limitations. First, although we adjusted for cardiovascular risk factors and comorbidities, we cannot rule out the possibility that unaccounted risk factors may be influencing the observed acceleration in epigenetic aging or the increased risk of brain health events. Second, our cross-sectional observational analysis is likely influenced by survival bias. It’s reasonable to assume that survivors of brain health events are generally healthier and may demonstrate slower epigenetic aging compared to non-survivors. This factor could potentially skew our results towards the null hypothesis.

In conclusion, our findings using high quality data from the Health and Retirement Study cohort establish robust, bidirectional associations between epigenetic aging and brain health events. We have established that a history of stroke, dementia, or late-life depression is not only associated with accelerated epigenetic aging but also that an advanced epigenetic age increases the likelihood of these conditions. Through Mendelian Randomization analyses, we provide strong evidence supporting the causal nature of these relationships. Overall, our study makes a significant contribution to the understanding of aging-related brain health. It underscores the critical role of epigenetic factors and opens new pathways for future research and observational applications, particularly in early risk assessment and intervention strategies.

## METHODS

### Study design

We conducted a 2-stage observational and genetic study nested within the HRS. Our goal was to investigate two different hypotheses: first, that persons who have survived brain health events, including stroke, dementia, and late-life depression, exhibit epigenetic age acceleration; and second, that those with accelerated epigenetic aging are at an elevated risk for subsequent brain health events. Both hypotheses were examined through a combination of observational and genetic analyses. To investigate the first hypothesis, we performed a nested cross-sectional analysis on HRS participants who had available DNA Methylation data. This allowed us to assess the association between survival from brain health events and epigenetic aging. To test the second hypothesis, we implemented a prospective cohort design using the same HRS group with available methylation data. This design enabled us to observe whether individuals with accelerated epigenetic aging were more likely to experience subsequent brain health events. The genetic analyses for both stages were conducted using one-sample Mendelian randomizations within the HRS cohort.

### The Health and Retirement study

The HRS is an ongoing, longitudinal study that is nationally representative of older adults in the United States. Its primary aim is to provide a comprehensive understanding of the health and economic circumstances associated with aging at both individual and population levels. The HRS sample was assembled in several waves of enrollment and data collection. The HRS sample was compiled through multiple phases of recruitment and data collection. The inaugural cohort, enrolled in 1992, included individuals born between 1931 and 1941 (who were then aged 51–61), along with their spouses of any age. Subsequently, a distinct study named “Asset and Health Dynamics Among the Oldest Old” (AHEAD) was conducted, focusing on the cohort born between 1890 and 1923 (who were then aged 70 and above). In 1998, these two samples were merged and supplemented with the addition of two more cohorts: the “Children of the Depression” (CODA, born 1924–1930) and the “War Babies” (born 1942–1947). This was done to ensure the sample accurately represented the U.S. population over the age of 50. Later, the “Early Baby Boomers” (EBB, born 1948–1953) and the “Mid Baby Boomers” (MBB, born 1954–1959) were added in 2004 and 2010, respectively. The most recent addition was the “Late Baby Boomers” (LBB, born 1960–1965) in 2016^56^. As of now, the HRS has successfully enrolled over 40,000 participants. Among these, nearly 20,000 have provided DNA samples, and DNA Methylation (DNAm) data has been obtained from 4,000 participants. The study conducts biennial interviews with participants, covering a broad range of variables such as income, employment, disability, physical health and functioning, and cognitive functioning. Further details about the HRS and its survey design can be found elsewhere^57^. The study’s protocol has received approval from the University of Michigan’s institutional review board, and informed consent has been obtained from all participants.

### Analytic sample

The present study utilized a subset of participants from the HRS who had available DNA Methylation (DNAm) data. DNAm assays were conducted on a non-random subsample of 4,018 individuals who took part in the Health and Retirement 2016 Venous Blood Study^58^. The sample is predominantly female (54.3%) with a median age of 66 years, and ages ranging from 50 to 100 years. The sample exhibits racial diversity with 10.0% being non-Hispanic Black, 8.9% Hispanic and 81.1% non-Hispanic White and others. The sample is also socioeconomically diverse as indicated by the educational distribution: less than high school (14.0%), high school/GED (29.9%), some college (25.8%), and college+ (30.3%). More than a third of the sample is obese (44.5%), 11.0% are current smokers, and 44.2% are former smokers. The sample has been weighted to ensure it is representative of the broader U.S. population^58^.

### DNA methylation data

Detailed information on the 2016 Venous Blood Study is provided in the VBS 2016 Data Description^58^. Blood samples were obtained from willing respondents during in-home phlebotomy visits, ideally scheduled within four weeks of the 2016 HRS core interview. Although fasting was suggested, it was not required. Methylation was assessed using the Infinium Methylation EPIC BeadChip. To ensure a balanced representation of key demographic variables (such as age, cohort, sex, education, and race/ethnicity), samples were randomized across plates, including 40 pairs of blinded duplicates. The correlation for all CpG sites was found to be greater than 0.97 when duplicate samples were analyzed. Data preprocessing and quality control were performed using the minfi package in R. A total of 3.4% of the methylation probes (equivalent to 29,431 out of 866,091) were excluded from the final dataset due to subpar performance, as determined by a detection p-value threshold of 0.01. Following the removal of these probes, samples that failed the detection p-value analysis were identified and removed using a 5% cut-off (minfi), resulting in the exclusion of 58 samples. Any samples that mismatched in sex and any controls (including cell lines and blinded duplicates) were also removed. High-quality methylation data were retained for 97.9% of the samples (n = 4,018). Any missing beta methylation values were replaced with the mean beta methylation value of the respective probe across all samples before the construction of DNAm age measures.

### Epigenetic clocks

Thirteen epigenetic clocks have been constructed using the HRS DNAm data. These clocks are calculated as a weighted sum of aging-related CpGs, typically ranging from 100 to 500, with weights determined using a penalized regression model. These methylation clocks, which represent epigenetic age, are measured in epigenetic years, with the premise that each tick of the clock signifies aging. Among these thirteen clocks, nine are classified as first-generation clocks, calibrated based on age^6–10,39,59–61^, while the remaining four are second-generation clocks, calibrated on health-related outcomes, namely Zhang^12^, PhenoAge^3^, GrimAge^11^, and MPOA^62^. These clocks exhibit significant variability in their mean values, ranges, and minimum and maximum ages. Some of the clocks, when expressed in years, have extremely high maximum ages (for example, Lin at 133 and Weidner at 148), while others have very low minimum ages (for example, Lin at 1.9). To create a composite value representing epigenetic age without any a priori selection of the clocks, we standardized them to approximate a normal distribution and took the average of these standardized clocks as our primary measure of epigenetic age. We also report results corresponding to each individual clock.

### Genetic data

The genotyping for this study was carried out by the Center for Inherited Disease Research in the years 2011, 2012, and 2015. Detailed information regarding quality control can be accessed in the online Quality Control Report^63^. Genotype data was collected from over 15,000 HRS participants using the Illumina HumanOmni2.5 BeadChips (HumanOmni2.5–4v1, HumanOmni2.5–8v1), which measures approximately 2.4 million SNPs. The Genetics Coordinating Center at the University of Washington, Seattle, WA, performed the genotyping quality control. Criteria for removal included individuals with missing call rates exceeding 2%, SNPs with call rates less than 98%, Hardy-Weinberg Equilibrium p-value less than 0.0001, chromosomal anomalies, and first-degree relatives in the HRS. Imputation to the 1000 Genomes Project Phase I v3 (released March 2012) was conducted using SHAPEIT2 and IMPUTE2. A worldwide reference panel consisting of all 1,092 samples from the Phase I integrated variant set was utilized. The Genetics Coordinating Center at the University of Washington, Seattle, WA, performed and documented these imputation analyses. All positions and names are aligned to the GRCh37/hg19 build.

### Genetic instruments

We utilized genetic instruments derived from external genome-wide association studies (GWASes) to represent the exposure variables: brain health events for the first stage and epigenetic age for the second stage.

#### 1^st^ stage

Our selection of genetic instruments involved the following sources for stroke, dementia and depression, respectively: the GIGASTROKE consortium’s GWAS of all-cause stroke^64^, the European Alzheimer & Dementia Biobank consortium’s GWAS of Alzheimer’s disease^65^, and a meta-analysis of the three largest GWASes of depression^66^. From each of these studies, we selected single nucleotide polymorphisms (SNPs) that were biallelic, common (minor allele frequency greater than 5%) and associated with the respective trait (p < 1e-5). To ensure the independence of these SNPs, we filtered out variants with an r2 (a measure of correlation between two genetic variants) greater than 0.1. This resulted in 382 SNPs for stroke, 256 for Alzheimer’s disease, and 462 for depression. These SNPs were combined to yield 1100 instruments associated with either stroke, Alzheimer’s disease, or depression. From this pool, 20 variants were excluded to ensure independence, 75 were not present in the imputed HRS genetic data, and 20 palindromic SNPs were excluded, resulting in a final list of 985 instruments. We then estimated the effect of the genetic instruments on the epigenetic age and on the brain health composite by conducting single-SNP association tests in HRS (Figure 2). The effect estimates corresponding to epigenetic age were obtained in HRS participants with DNAm and genetic data and the ones corresponding to brain health events were obtained in all HRS participants with genetic data (Figure 1).

#### 2^nd^ stage

For the second stage, we selected genetic instruments by combining data from multi-ethnic GWASes^67^ of six epigenetic clocks: GrimAge^11^, Hannum^8^, PhenoAge^3^, Horvath^9^, PAI-1^11^, and Gran^3,11,40^. From each of these GWASes, we selected common SNPs (minor allele frequency >5%) associated with the respective epigenetic clock (p < 1e-5). To ensure the independence of these SNPs, we filtered out variants with an r2 greater than 0.1. This yielded 81 SNPs for the GrimAge clock, 84 for the Hannum clock, 104 for the PhenoAge clock, 103 for the Horvath clock, 75 for the PAI-1 clock, and 403 for the Gran clock. These SNPs were combined to obtain a pooled list of 850 SNPs associated with any of the six epigenetic clocks. From this pool, 52 variants were excluded to ensure independence, 6 were not present in the imputed HRS genetic data, and 15 palindromic SNPs were excluded, resulting in a final list of 777 instruments. We then estimated the effect of the genetic instruments on the epigenetic age and on the brain health composite by conducting single-SNP association tests in HRS (Figure 3).

### Ascertainment of brain health events

#### Stroke

Stroke events were identified as the first instance of stroke in a dedicated variable evaluated throughout the study period (1992–2020), based on self-reported or proxy-reported doctor’s diagnosis (Has a doctor ever told you that you had a stroke?). In cases where participants were unable to be directly interviewed (e.g., deceased), health care proxies were interviewed. Transient ischemic attacks were not systematically assessed and were not classified as strokes, and information on stroke subtype was not available. Previous studies using HRS data have demonstrated that associations between known risk factors and self-reported stroke incidence in the HRS align well with associations in studies using observationally verified strokes^68^. Moreover, self-reported strokes in the HRS corresponded well with strokes coded according to the International Classification of Diseases in the Centers for Medicare and Medicaid Services records, with a sensitivity of 74% and a specificity of 93%^69^.

#### Dementia

The ascertainment of all-cause dementia among self-respondents was carried out at each wave using the modified version of the Telephone Interview for Cognitive Status (TICS): a 27-point cognitive scale that encompasses immediate and delayed 10-noun free recall tests (each with a range of 0–10 points), a serial seven subtraction test (range: 0–5 points), and a backward count from 20 test (range: 0–2 points)^70,71^. Based on their continuous score, we categorized cognitive status into two groups—those with and without dementia—using observationally verified cutpoints from the Aging, Demographics, and Memory Study (ADAMS). A supplemental study of the HRS, ADAMS involves in-home neuropsychological and observational assessments combined with expert clinician adjudication to obtain a gold-standard diagnosis of cognitive status^70,72^. Respondents with scores ranging from 12 to 27 were classified as non-impaired; those with scores from 7 to 11 were identified as having cognitive impairment but no dementia; and those with scores from 0 to 6 were classified as having dementia. For the purposes of this paper, we focused solely on participants with dementia. A small percentage of respondents (0.8%–3.1%) declined to participate in tests of immediate and delayed recall and serial 7s. To address this, HRS has developed an imputation strategy for cognitive variables across all waves^73^.

#### Late-life depression

Following a common definition from the literature^74–77^, we defined late-life depression as a major depressive episode occurring after the age of 65 in an individual with no history of depressive episodes prior to this age. Depressive symptoms were evaluated using the validated, modified 8-item version of the Center for Epidemiologic Studies-Depression (CES-D) scale^78,79^. During each biennial questionnaire, participants were asked to indicate (yes/no) whether they had experienced any of the 8 symptoms in the preceding week. A summary score (ranging from 0 to 8) was compiled by adding the number of affirmative responses across the 8 items, with two positively framed items being reverse-coded^78^. Major depressive episodes were identified using dichotomized CES-D summary scores for each wave, with a cutoff of ≥ symptoms. This threshold has been previously validated and is considered equivalent to the 16-symptom cut-off of the well-validated 20-item CES-D scale^76,78,80^. In our sensitivity analyses, we explored an alternative definition of late-life depression found in the literature, characterized by a lower age cutoff of 60 years, instead of 65^81–83^.

### Covariates ascertainment

We collected self-reported demographic and socioeconomic variables at the onset of the Venous Blood Study^58^, including age (continuous), sex (male or female), and race and ethnicity (non-Hispanic White, non-Hispanic Black, Hispanic or other). Additionally, we gathered self-reported measures of health behaviors and health conditions at baseline, such as body mass index (continuous, kg/m2 derived from self-reported height and weight), and cigarette smoking status (nonsmoker, former smoker, current smoker). Health conditions were determined based on responses (yes/no) to the question “Has a doctor ever told you that you had a (health condition)?” for heart disease, diabetes, and hypertension. Previous studies using HRS data have shown that self-reported health conditions align substantially with medical records data, and that the self-reported health behavioral measures have strong external validity^84–88^.

### Statistical analyses

We describe discrete data as counts (percentages) and continuous data as mean (standard deviation) or median (interquartile range), as appropriate. In the first stage of the study, which examined the association between a history of brain health events (exposure) and epigenetic age (outcome), a history of brain health events was defined as having experienced a stroke, dementia, or late-life depression episode ascertained in waves 1 (1992) to 13 (2016). In the second stage of the study, which examined the association between epigenetic age (exposure) and the onset of new brain health events (outcomes), these events were defined as a stroke, dementia, or late-life depressive episode ascertained in waves 14 (2018) or 15 (2020). Participants who did not participate in both of these waves, due to loss to follow-up or death, were excluded from this analysis. Additionally, participants who had experienced brain health events between waves 1 and 13 were also excluded from this phase of the analysis.

In the first stage of our study, we explored the association between a history of brain health events and epigenetic age using multivariable linear regression models. These models were either unadjusted (Model 1), adjusted for potential demographic confounders such as age, sex, and race/ethnicity (Model 2), or adjusted for these demographic factors and cardiovascular risk factors (hypertension, diabetes, smoking, and body mass index), and comorbidities (history of heart events including heart attack, coronary artery disease, angina, and congestive heart failure, Model 3). In the second stage, we investigated the association between epigenetic age and the risk of new brain health events using multivariable logistic regression models. These models were either unadjusted (Model 1) or adjusted for the same sets of confounders as in the first stage (Model 2 and 3).

#### Mendelian Randomization

In both stages, our primary MR analyses used the inverse variance weighted (IVW) method. In secondary analyses, we tested for horizontal pleiotropy (the possibility that the effect of the instrument on the outcome of interest is exerted through a pathway other than the exposure) using the Mendelian Randomization Pleiotropy Residual Sum and Outlier (MR-PRESSO^89^) global test with 10,000 simulations and the MR-Egger intercept term^90^. To account for this possible phenomenon, we implemented the weighted median method, a robust alternative to the IVW method that allows for up to 50% of the genetic variants used to be invalid instrumental variables without biasing the causal effect estimate^91^. Additionally, the weighted median approach is less sensitive to outliers than the IVW method, which can be useful in the presence of genetic variants with extreme effect estimates^26^.

#### Secondary and sensitivity analyses

In our secondary analyses, we repeated the epidemiological analyses for both stages, considering each brain health outcome individually (stroke, dementia, and depression), as well as a composite outcome that included only stroke and dementia. In addition to our main measure, the mean epigenetic age, we also report the association results for each epigenetic clock. In our sensitivity analyses, we: (1) tested the association between epigenetic age and the risk of new brain health events, excluding only participants missing data for waves 14 or 15, as opposed to excluding participants missing both waves; (2) repeated both stages using an age cutoff of 60 to ascertain late-life depression.

#### Software

Statistical analyses were performed using R 4.2.1^92^ and the following packages: dplyr, ggplot2, ggforestplot, tableone, TwoSampleMR, MR-PRESSO, gwasvcf, ieugswar.The current manuscript is written in line with the STROBE (Strengthening the Reporting of Observational Studies in Epidemiology) guidelines (Supplementary Table X).

## Figures and Tables

**Table 1.** Cohort characteristics

Variable	Overall (n=4018)	Prevalent brain health events (n=806)	Incident Brain health events (n=261)
**Demographics**			
**Age (mean (SD))**	69.9 (9.6)	75.2 (10.1)	73.0 (9.3)
**Male gender**	1669 (41.5)	334 (41.4)	115 (44.1)
**Race**			
**White**	3013 (75.0)	572 (71.0)	204 (78.2)
**Black**	674 (16.8)	170 (21.1)	40 (15.3)
**Hispanic**	207 (5.2)	42 (5.2)	12 (4.6)
**Other**	122 (3.0)	22 (2.7)	5 (1.9)
**Cardiovascular Risk factors**			
**Prevalent hypertension**	2559 (63.7)	604 (74.9)	186 (71.3)
**Prevalent diabetes**	1151 (28.6)	306 (38.0)	85 (32.6)
**BMI (mean (SD))**	28.92 (6.30)	28.4 (6.4)	28.9 (6.1)
**Smoking**			
**Past**	1776 (44.2)	399 (49.5)	118 (45.2)
**Never**	1764 (43.9)	307 (38.1)	113 (43.3)
**Current**	455 (11.3)	95 (11.8)	29 (11.1)
Prevalent heart condition*	1098 (27.3)	356 (44.2)	80 (30.7)

* Heart conditions include: heart attack, coronary artery disease, angina, congestive heart failure

Note: The terms prevalent, respectively incident, refer to conditions having occurred before, respectively after, the epigenetic age estimation performed during the 2016 wave.

**Table 2.** Multivariable regression results: changes in mean epigenetic age following a brain health event and odds ratios of brain health events per one standard deviation increase in mean epigenetic age

	1^st^ stage			2^nd^ stage	
Outcome	Change in mean epigenetic age as a function of prevalent brain health events			Change in mean odds of incident brain health events as a function per 1 standard deviation increase in mean epigenetic age	
Statistical model	Linear regression			Logistic regression	
Covariates	% change	Beta (SE)	P	Odds Ratios (95% CI)	P
**Unadjusted Model 1**	42%	0.42 (0.02)	<0.001	2.62 (2.10–3.25)	<0.001
**Multivariable Model 2**	8%	0.08 (0.01)	<0.001	1.70 (1.16–2.50)	0.007
**Multivariable Model 3**	4%	0.04 (0.01)	0.002	1.48 (0.99–2.21)	0.057

Model 2: Adjusted for age, sex and race/ethnicity

Model 3: Adjusted for age, sex, race/ethnicity, hypertension, diabetes, smoking, BMI, history of heart attack, coronary artery disease, angina, or congestive heart failure

**Table 3.** Mendelian Randomization analyses.

Analytical approach for Mendelian Randomization analyses	1^st^ stage			2^nd^ stage		
	Genetically modeled exposure = Risk of brain health events			Genetically modeled exposure = Epigenetic age		
	Outcome = Epigenetic age			Outcome = Risk of brain health events		
	Number of instruments	Beta (SE)	P	Number of instruments	OR (95% Cl)	P
**Primary IVW MR**	985	0.11 (0.03)	<0.001	777	1.15 (1.06 – 1.25)	<0.001
**Weighted median MR**	985	0.08 (0.04)	0.052	777	1.15 (1.00 – 1.31)	0.048
**MR-Egger**	985	0.10 (0.04)	0.01	777	1.15 (1.00 – 1.31)	0.047

Abbreviations: IVW = Inverse probability weighted; MR = Mendelian Randomization; SE = Standard error; CI = confidence interval; OR = odds ratio.

## Data Availability

The data used in this study can be accessed by contacting the Health and Retirement Study (https://hrs.isr.umich.edu/about).
